# The Role of Platelet-Rich Plasma (PRP) in the Treatment of Patellofemoral Arthritis and Anterior Knee Pain: A Systematic Review

**DOI:** 10.3390/ijms26189006

**Published:** 2025-09-16

**Authors:** Byron Chalidis, Charalampos Pitsilos, Vasileios Davitis

**Affiliations:** 1 1st Orthopaedic Department, Aristotle University of Thessaloniki, 57010 Thessaloniki, Greece; 2 Academic Department of Trauma and Orthopaedics, School of Medicine, University of Leeds, Leeds LS1 3EX, UK; chpitsilos@outlook.com; 3 2nd Orthopaedic Department, Aristotle University of Thessaloniki, 54635 Thessaloniki, Greece; vasilisdavitis@hotmail.com

**Keywords:** platelet-rich plasma, patellofemoral osteoarthritis, anterior knee pain, chondromalacia patellae, intra-articular injection

## Abstract

Patellofemoral osteoarthritis (OA) and chondromalacia patellae (CMP) are common and disabling conditions that significantly affect physical performance and quality of life. Despite the great deal of scientific research on the subject, there is limited evidence regarding the outcome of nonoperative interventional procedures. Platelet-rich plasma (PRP) has demonstrated positive results for tibiofemoral knee osteoarthritis, but its role in anterior knee pain (AKP) remains unclear. The aim of this study was to review the evidence on the efficacy (clinical and radiological) and safety of PRP in patients suffering from patellofemoral OA, CMP, and AKP. Medline/Pubmed, Web of Science, and Scopus databases were systematically searched up to June 2025 to identify all the available relevant studies. Five studies, including 146 patients, fulfilled the eligibility criteria and were included in the systematic review. Although there was a statistically significant improvement in clinical setting, radiologic evidence of cartilage regeneration was limited and uncertain. Specifically, the pooled analysis revealed an improvement of the Visual Analogue Scale from 6.7 to 2.1 (*p* < 0.001), the Western Ontario and McMaster Universities Osteoarthritis Index score from 24 to 10.3 (*p* < 0.001), the Oxford score from 35.1 to 37.4 (*p* < 0.001), the Kujala score from 71 to 83 (*p* < 0.001), and the Tegner/Lysholm score from 65.3 to 86.5 (*p* < 0.001). Well-designed and appropriately powered randomized trials with imaging endpoints are needed to validate the efficacy of PRP administration in PFA, CMP, and AKP and refine patient selection criteria.

## 1. Introduction

Osteoarthritis (OA) is a leading cause of disability, affecting over 500 million people worldwide and imposing a major socioeconomic burden. It is a multifactorial disease influenced by occupational stress, high-impact sports, prior injuries, obesity, and sex-related differences. In the knee joint, early diagnosis is crucial for timely intervention and management, which can significantly improve patient satisfaction and outcomes. Advance radiological imaging with MRI is often required to assess cartilage integrity and degeneration, as reliance on subjective clinical assessment and plain radiographs often underestimate early cartilage damage [1]. The condition may affect only the anterior compartment of the knee causing pain and discomfort particularly during squatting, deep sitting, or climbing stairs.

Anterior knee pain (AKP) is a prevalent condition with a negative impact on quality of life, particularly in the young adult female population, where it can affect up to 27% of individuals [2]. The disorder encompass a heterogeneous spectrum of clinical manifestations arising from different origins. However, the phrase “anterior knee pain” is now considered a general descriptive term unrelated to the etiology and nature of the underlying lesion [3,4].

Chondromalacia patellae (CMP) is one of the most common causes of chronic knee pain that is characterized by softening and swelling of the patellar cartilage. The condition can progress to advanced cartilage degeneration and osteoarthritis (OA), if left untreated. However, due to its nonspecific nature and lack of clarity regarding the underlying cause, the term “chondromalacia patellae” has largely been abandoned in clinical use. Patellofemoral OA is another frequent anterior knee disorder that refers to degenerative cartilage changes in the patellofemoral compartment, involving the trochlear groove and the posterior patellar surface. This condition may develop as a primary degenerative disease, often linked to age-related wear, joint malalignment, ligamentous laxity, or as a secondary condition following trauma. Post-traumatic patellofemoral arthritis (PFA) is usually a result of childhood patellar dislocation and cartilage injury, which may remain asymptomatic for a long period of time until early adulthood. In contrast, primary patellofemoral OA tends to develop insidiously and is commonly associated with biomechanical factors that impairs patellofemoral alignment and stability [5], although it represents a distinct clinical and radiological entity it may coexist with medial and lateral tibiofemoral arthritis. According to a radiographic study by Duncan et al. [6], the most frequent knee OA pattern involved both patellofemoral and tibiofemoral compartments (40%), followed by isolated patellofemoral OA (24%), and isolated tibiofemoral OA (4%). Other epidemiological research studies by Davis et al. [7] and McAlindon et al. [8] further revealed that isolated patellofemoral joint OA has a prevalence of 8–9% showing a higher incidence in females (24%) than males (11%).

First-line management of osteoarthritis includes oral nonsteroidal anti-inflammatory drugs, exercise therapy, weight reduction for those who are overweight, patient education, and self-management programs designed to help individuals take control of their condition. For patellofemoral pain, a combination of knee strengthening exercises with foot orthoses or patellar taping is advised [9]. Platelet-rich plasma (PRP) is an autologous blood derivative with a supraphysiological concentration of platelets, rich in bioactive molecules that exert regenerative and anti-inflammatory effects [10,11]. Platelet-rich plasma has widespread application in orthopedics, particularly in the management of knee OA [12]. Its efficacy is attributed to high levels of growth factors—such as platelet-derived growth factor, transforming growth factor-beta, and vascular endothelial growth factor—that stimulate chondrocyte proliferation, extracellular matrix synthesis, and tissue repair [13]. There is growing clinical evidence for its efficacy for the treatment of knee cartilage lesions and OA and the available comparative clinical trials have demonstrated superior clinical outcomes of the application of PRP over hyaluronic acid (HA) or cortisone injections [14,15,16]. However, despite extensive research in the field of global knee OA, there is scarce and heterogeneous evidence regarding the efficacy of PRP in patellofemoral joint degeneration and AKP.

Therefore, we conducted a systematic review to evaluate the available evidence regarding the outcome of PRP knee injections in PFA and AKP and compare its efficacy with the other types of non-operative treatment. Furthermore, we focused on the molecular mechanisms that may underlie its clinical effect and benefit.

## 2. Materials and Methods

### 2.1. Protocol and Registration

The present systematic review was conducted following the Preferred Reporting Items for Systematic Review and Meta-Analysis (PRISMA) guidelines [17]. The review protocol was registered in the International Prospective Register of Systematic Reviews (PROSPERO) with a specific registration number CRD420251107031.

### 2.2. Search Strategy

A comprehensive literature search was performed by two investigators (V.D., C.P.), using the Medline (Pub-Med), Web of Science, Scopus, and Cochrane Central databases up to 30 June 2025. A third researcher (B.C.) helped to resolve any disagreements and provide a consensus. Firstly, the titles and abstracts of the identified studies were assessed for eligibility, followed by full-text articles examination. The search strategy incorporated both Medical Subject Headings (MeSH) and free-text terms including: “platelet-rich plasma”, “PRP”, “anterior knee pain”, “patellofemoral arthritis”, “chondromalacia patella”, and “patellofemoral chondropathy”. Boolean operators such as “AND” and “OR” were applied to refine the results. The review was limited to studies published in the English language and involving human subjects. Moreover, the references of the included articles were also scrutinized manually to identify any omitted eligible studies.

### 2.3. Eligibility Criteria

Studies including patients with PFA and AKP who received intra-articular PRP injections and reported the outcome of this application were enrolled for further evaluation. The eligibility criteria included clinical research human studies (randomized controlled trials, prospective or retrospective cohort studies, and case series) which were published in English language journals and reported the effect of intra-articular injections of PRP in patients suffering from PFA and subsequent AKP. Non-original articles (e.g., reviews, editorials, letters to the editor), case reports, animal or in vitro studies, and studies without separate analysis of PFA were excluded from further evaluation.

### 2.4. Data Extraction and Analysis

Data were extracted and recorded from each included study is as follows: first author, year of publication, study type, age, gender, sample size (number of patients and/or number of knees), PRP preparation and injection protocol, follow-up duration, and adverse events. The collected outcome measures included the Visual Analogue Scale (VAS) for pain, Kujala Score, Oxford Knee Score, Tegner and Lysholm scores, and the Western Ontario and McMaster Universities Osteoarthritis Index (WOMAC) score. Where applicable, imaging findings and particularly T2-mapping MRI were also recorded and considered secondary endpoints of the study. A pooled analysis and meta-analysis of clinical outcomes were deemed inappropriate due to the heterogeneity and potential risk of bias among the included studies.

### 2.5. Level of Evidence and Quality of Studies

Based on the study type, different tools were used for the assessment of the risk of bias. The risk of bias 2 (RoB 2) tool was used for the assessment of randomized control trials [18] and the methodological index for non-randomized studies (MINORS) was used for non-randomized studies, comparative or not [19]. These tools allow the rater to assign a three-level quality score—“good”, “moderate”, or “poor”. Good quality indicates “low” risk of bias, moderate quality “some concerns/moderate” risk of bias, and poor quality “high” risk of bias. The size effects (statistical power of the minimum sample sizes) were calculated as follows: 90% for 34 cases, 80% for 26 cases, 70% for 21 cases and 60% for 17 cases [20].

### 2.6. Statistical Analysis

Once the data extraction was completed, the collected data were transcribed into SPSS (IBM Corp., Armonk, NY, USA, released 2017; IBM SPSS Statistics for Windows, Version 25.0) and analyzed. For each study, mean difference (MD) between baseline and follow-up were calculated, and standard errors were derived from reported or estimated standard deviations. Pooled estimates were obtained using both fixed-effect and random-effects models. Heterogeneity was assessed using Cochran’s Q test. A 95% confidence interval (CI) was used. Statistical significance was assumed at a *p* value of less than 0.05.

## 3. Results

### 3.1. Literature Search and Study Identification

The initial literature search identified 734 potentially relevant articles. After duplicate removal and the screening of titles and abstracts, 696 articles were excluded as irrelevant. The full text of the remaining 38 articles was then reviewed in detail. Of these, 33 were excluded due to reasons such as review or case report article type or lack of specific data concerning PFA or AKP. Ultimately, 5 clinical studies fulfilled all predefined inclusion criteria and were selected for systematic review.

**Figure 1:**
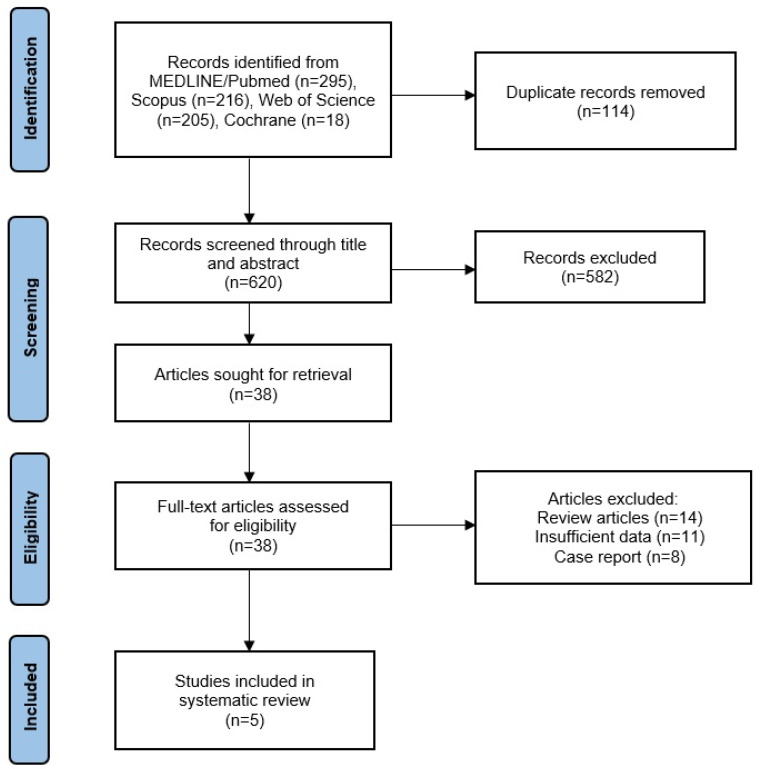
PRISMA flow diagram for search results.

The selected articles investigated the outcome of PRP treatment on patients suffering from AKP due to PFA and CMP. They consisted of two prospective cohort studies, two retrospective cohort studies, and one randomized controlled trial. Four studies incorporated a comparison group whose participants received conservative treatment, physiotherapy, and prolotherapy. All studies reported quantitative outcomes related to pain, knee function, or imaging. The process of study identification, screening, eligibility assessment, and inclusion of relevant studies was performed according to the PRISMA guidelines, as depicted in the PRISMA flow diagram (Figure 1) and summarized in the accompanying tables.

### 3.2. Quality Assessment and Risk of Bias of the Eligible Studies

All studies were assessed to have some concerns/moderate risk of bias. The level of evidence, the methodological quality, the risk of bias, and the size effects of the included studies are described in Table 1.

### 3.3. Characteristics of the Eligible Studies and Patients

In total, 146 patients and 148 knees were included across the five eligible studies [21,22,23,24,25]. The mean reported age of the participants ranged from 18 to 57 years. The follow-up periods in these studies varied between 3 months and at least 12 months. The included studies were designed as a retrospective cohort study, prospective double-blind randomized controlled trial, single-center prospective cohort study, retrospective observational study, and non-randomized controlled trial. All studies enrolled adult patients complaining of AKP due to clinically and/or radiologically confirmed chondromalacia patella and patellofemoral osteoarthritis that were refractory to conservative treatment options. PRP protocols were relatively homogeneous with most studies delivering three intra-articular injections spaced one to three weeks apart [21,23,24]. In three studies PRP monotherapy was applied [21,22,23]. In the remaining two studies the PRP was combined with injection of mesenchymal stem cells (MSC) [25] or hyaluronic acid [24]. Patient demographics, study types, and treatment details are summarized in Table 2. The participants typically complained of chronic AKP due to PFA or chondropathy that was confirmed clinically and radiologically. In all the included studies, patients with prior knee surgery, severe comorbidities, or incomplete follow-up were excluded. The risk of bias was minimized by randomization or blinding in most studies and by consistent reporting of adverse events, which were rare or not observed.

### 3.4. Injections Type, Doses, Frequency, and Site of Injection

Platelet-rich plasma protocols were relatively homogeneous, with most studies delivering three intra-articular injections spaced one to three weeks apart [21,23,24]. In three studies, PRP monotherapy was applied [21,22,23]. In the remaining two studies the PRP was combined with MSC [25] or HA [24]. The PRP was consistently prepared from autologous blood, though specific preparation details, dose, and administration protocols varied among studies. Doses of PRP ranged from 4 to 10 mL per injection. Gürsoy et al. [22] administered 5 cc per injection; Bellisari et al. [21] used 8–10 mL per injection; Ostojic et al. [24] reported 4–6 mL per injection; some studies did not specify the exact dose. The intra-articular injections were always carried out into the affected knee via a superolateral patellar approach [22], using or not using ultrasound [22,23] or fluoroscopic--guided techniques [25]. In all studies, the procedure was performed under aseptic conditions, typically by an experienced clinician or orthopedic specialist.

### 3.5. Outcomes, Adverse Effects, and Protocol Heterogeneity

All five included studies demonstrated that PRP treatment led to clinically meaningful improvements in pain and/or function in symptomatic patients with PFA and chondromalacia patella. In a prospective cohort study by Pintat et al. [25], 19 patients with patellofemoral osteoarthritis received a single intra-articular injection with PRP and MSC. Although significant improvement in WOMAC scores, from a mean of 34.3 at baseline to 14.1 at 12 months post-injection was reported (*p* < 0.0018), quantitative MRI T2 mapping did not show statistically significant changes in cartilage grade, surface area, or T2 relaxation time (*p* > 0.375). Notably, no adverse events or complications were described throughout the study period. In the retrospective cohort study by Cobianchi Bellisari et al. [21], 34 patients received three leukocyte-reduced PRP injections at three-week intervals. At the six-month follow-up, patients experienced a substantial reduction in pain (VAS: 7.0 to 2.0, *p* < 0.001) and improved function (WOMAC: 18.3 to 7.3, *p* < 0.001). Importantly, MRI T2 mapping demonstrated a significant decrease in global cartilage T2 values (from 44.2 ms to 41.5 ms, *p* < 0.001), indicating potential improvement in cartilage ultrastructure, along with a reduction in T2 values for focal cartilage lesions (from 70.1 ms to 59.9 ms, *p* < 0.001). In contrast, the control group treated with conservative modalities exhibited no significant change in T2 mapping values. In another non-randomized cohort study, Ostojic et al. [24] evaluated 43 patients with AKP that were treated with three PRP injections and one hyaluronic acid injection. Statistically significant improvements in both Kujala and VAS pain scores at six months were reported compared to the physiotherapy-alone group. Younger age was correlated with greater improvements in pain and function. In addition, no serious adverse events or complications were reported. In the retrospective cohort study by Gürsoy et al. [22], 27 patients with AKP and positive Hoffa’s fat pad impingement tests received two PRP injections, demonstrating marked improvements in VAS (7.1 to 4.1), Lysholm (66.6 to 78.2), and Oxford Knee scores (35.1 to 37.4) at six months. MRI showed a slight increase in Hoffa fat pad volume post-PRP administration, but this finding was not correlated with an improvement in clinical outcome. Finally, the double-blind randomized controlled trial by Örsçelik et al. [23] compared PRP with prolotherapy in 69 patients with chondromalacia patellae. PRP resulted in greater improvements in VAS (7.5 to 1.0) and Lysholm scores (64.1 to 94.5) at 12 months compared to prolotherapy. However, no MRI follow-up data were provided.

No serious adverse events were reported across all studies and PRP injections were well tolerated by all patients. Mild and transient local swelling or discomfort was occasionally observed but did not affect the overall outcome. Protocol heterogeneity was noted among studies, including differences in PRP preparation, number and time interval of injections, and simultaneous intra-articular administration or not of MSCs or HA substances. While most studies utilized leukocyte-reduced PRP, the exact preparation process varied. Furthermore, the follow-up ranged from six to twelve months. Despite these methodological differences, the evidence from these five studies supports the efficacy and safety of PRP for the management of symptomatic PFA, CMP, and AKP. However, the limited data highlights the need for further research to establish standardized protocols and optimal patient selection.

A pooled analysis of the outcome of PRP on functional scores was performed. The data of the included studies are summarized in Table 3. The VAS score was measured in four studies [21,22,23,24], including 127 patients. The mean value was reduced from 6.7 to 2.1 at the final follow-up (*p* < 0.001). The pooled mean difference (MD) was −4.7 (95% CI −5.6 to −3.8) under the fixed-effect model and −4.4 (95% CI −6.6 to −2.2) under the random-effects model. However, heterogeneity was very high (I^2^ ≈ 98%). The WOMAC score was evaluated in two studies [21,25] with 53 patients. A statistically significant improvement was found, from 24 to 10.3 (*p* < 0.001). The MD was found −11.2 (95% CI −13.0 to −9.4) under fixed-effects and −14.4 (95% CI −22.6 to −6.2) under random-effects. Heterogeneity was moderate (I^2^ ≈ 72%). The Oxford and the Kujala scores were measured in one study each [22,24], with 27 and 28 patients, respectively. The Oxford score improved from 35.1 to 37.4 (*p* < 0.001) and the Kujala score increased from 71 to 83 (*p* < 0.001). In two studies [22,23] with a total number of 65 patients, the Tegner/Lysholm was evaluated. The mean value was improved from 65.3 at baseline to 86.5 at the final follow-up (*p* < 0.001). The MD was +22.6 (95% CI +21.1 to +24.1) under fixed-effect and +21.0 (95% CI +15.0 to +27.1) under random-effect models, again with very high heterogeneity (I^2^ ≈ 98%).

## 4. Discussion

Patellofemoral arthritis is recognized as a prevalent and clinically significant subset of knee arthritis, with isolated PFA affecting 8–24% of the general population and occurring more frequently in women [5]. The patellofemoral joint is a critical source of pain and functional impairment, especially during activities that load the joint (e.g., stair climbing and squatting) [26]. Etiological contributors include malalignment, muscular dysfunction, trochlear dysplasia, patella alta, prior trauma or instability, postoperative complications, and altered biomechanics, all of which promote joint stress and disease progression [5,27,28]. Initial management is non-operative and centers on patient education, weight reduction, physiotherapy, taping, bracing, and orthotics [26,27]. Physiotherapy, including hip and quadriceps strengthening, patellar mobilization, and taping, is supported by evidence for managing patellofemoral pain, though benefits may diminish over time [29]. Pharmacological options such as NSAIDs and duloxetine are used for symptomatic relief, whereas corticosteroid injections are less favored due to potential chondrotoxicity [30].

For persistent cases, injection therapies are increasingly utilized in clinical practice. Viscosupplementation with hyaluronic acid offers transient symptom relief but lacks disease-modifying effects [5]. In contrast, regenerative and orthobiologic interventions—including PRP, prolotherapy, and stem cell therapies—are gaining traction, with mounting evidence supporting the efficacy of PRP and in some studies its superiority over prolotherapy [30,31]. In the literature, intra-articular application of PRP demonstrated a favorable safety profile and its capacity to improve pain, function, and quality of life in PF lesions has been emphasized and has gained increasing attention [32]. Adipose-derived stem cells and bone marrow aspirate concentrate also have emerged as safe and potentially effective treatment modalities for cartilage regeneration in refractory cases [31,32]. Surgical intervention is reserved for cases unresponsive to conservative and biologic therapies, including arthroscopic debridement, tibial tubercle osteotomy, and patellofemoral arthroplasty in patients with severe isolated PFA [5].

This systematic review demonstrates that intra-articular platelet-rich plasma injections, either as a standalone intervention or in combination with HA or MSC, are associated with significant pain reduction and clinical improvement in patients with AKP due to CMP and PFA. Across a range of study designs, populations, and PRP preparation protocols, a consistent trend of clinical benefit was observed. The available evidence indicates that validated outcome measures for pain (Visual Analogue Scale) and function (Lysholm, WOMAC, Kujala, and Oxford Knee scores) can be significantly improved after PRP administration. For example, Örsçelik et al. [23] reported a statistical superiority of PRP over prolotherapy. Specifically, the VAS pain score was reduced from 7.5 to 1.0 and the Lysholm score increased from 64.1 to 94.5 after one year of PRP injection. Similarly, Cobianchi Bellisari et al. [21] observed significant improvements in the WOMAC score (from 18.3 ± 4.5 to 7.3 ± 3.2) and VAS pain score (from 7 to 2), which were statistically greater compared to control group. Gürsoy et al. [22] also reported both a notable reduction in VAS pain score from 7.1 ± 0.9 to 4.1 ± 1.8 and a marked improvement in Lysholm and Oxford scores following PRP treatment for AKP. Additionally, Ostojic et al. [24] demonstrated that PRP combined with HA provided greater improvement in both pain and function at three and six months compared to controls. In the same study, the injection group yielded superior Kujala scores over conservative treatment with physiotherapy alone.

Advanced MRI sequences, including T2 mapping, further support the clinical efficacy of PRP in PF lesions. Cobianchi Bellisari et al. [21] demonstrated a statistically significant reduction in T2 relaxation times in PRP-treated patients indicating improved cartilage matrix integrity. Modified WORMS score decreased after treatment (from 14 ± 3.4 to 12.5 ± 2.4) with a mean improvement of 10.5%. In the majority of studies, the WORMS score was the preferred tool for evaluating the bone marrow lesions and served as a predictive marker for cartilage loss [33]. However, not all studies found any evidence of structural cartilage repair after a PRP injection [21]. For example, Pintat et al. [25] observed a significant improvement in the WOMAC score (from 34.3 to 14.1 at 12 months) after PRP plus MSCs injection, but MRI T2 mapping did not illustrate a significant difference in the grade of cartilage lesions. The authors emphasized that clinical improvements might precede or occur independently of measurable radiological changes. The role of imaging biomarkers was also examined by Gürsoy et al. [22], who evaluated any changes in Hoffa fat pad volume on MRI examination. Although PRP treatment led to an increase in fat pad volume and conventional therapy led to a decrease in fat pad volume, this difference was not correlated with the clinical outcome. The authors advocated that the clinical benefit after PRP injection may occur through mechanisms unrelated to fat pad volume changes. Nevertheless, there is still a lack of MRI studies to provide imaging evidence regarding the efficacy of PRP in the treatment of PFA and CMP. Moreover, there is no consensus regarding whether standard or advanced sequences such as T2 mapping should be administered for the evaluation of patellofemoral joint cartilage lesions [34]. Some studies have reported changes in T2 mapping after PRP treatment, but the results are generally inconclusive and highlight the necessity for establishing standardized imaging-based endpoints. This inconsistency between clinical benefit and imaging outcome suggests that the therapeutic effects of PRP may primarily derive from anti-inflammatory and joint environment-modifying mechanisms rather than from structural cartilage regeneration, which limits its recognition as a truly disease-changing intervention.

Regarding the safety of the method, no significant adverse events were reported in any of the included studies, supporting the conclusion that intra-articular PRP injection—alone or in combination with HA or MSCs—is a safe treatment modality for appropriately selected patients suffering from CMP, PFA, or AKP. The reviewed studies strictly adhered to the PRP preparation process and administration protocol. Örsçelik et al. [23] used 72 mL of autologous blood to yield 7 mL of PRP for each of three ultrasound-guided intra-articular injections at three-week intervals. Similarly, Cobianchi Bellisari et al. [21] utilized three intra-articular PRP injections (8–10 mL per session) at three-week intervals. The PRP was activated by calcium chloride and patients were advised to limit activity post-injection. Ostojic et al. [24] administered three PRP injections (4–6 mL each) in combination with HA at 7–10-day intervals. Patients were recommended to cease taking NSAIDs medication and increase hydration before PRP treatment. Gürsoy et al. [22] employed a protocol of two intra-articular injections, 14 days apart. The PRP obtained from 20 mL of venous autologous blood and injected into the symptomatic knee via the superolateral patellar approach. According to study results, PRP treatment reduced pain and knee swelling, improved patients’ quality of life, and outperformed hyaluronic acid in terms of clinical outcomes at six months [35].

Despite these positive outcomes, the efficacy of PRP in treatment of PF lesions remains subject to debate due to substantial heterogeneity in study design, PRP preparation, and administration protocols. A high degree of variability exists in PRP classification (pure PRP vs. leukocyte-rich PRP), platelet concentration, activation methods (e.g., thrombin, calcium chloride, mechanical trauma), and centrifugation protocols (single vs. double spin, manual vs. automated systems), all of which can affect platelet yield and growth factor content [36]. Furthermore, the lack of standardization in preparation techniques (e.g., centrifugation, platelet/leukocyte content) and injection methods (dose, cycles, activation), can lead to significant variation in outcomes and limits reproducibility and guideline support. Such heterogeneity in PRP type, concentration, activation, and dosing schedule complicates comparison across studies, limits the strength of conclusions, and highlights the urgent need for international standards in PRP preparation and administration.

The findings of this review are consistent with the existing literature regarding the effectiveness of PRP in knee osteoarthritis. Filardo et al. [37] observed that clinical improvements following PRP knee injections in patients suffering from knee degeneration are more evident at 6 to 12 months post-intervention. Many studies found PRP to be superior to hyaluronic acid [38,39,40] or saline solution [41] in terms of pain and function at the initial stages of knee osteoarthritis. Sustained benefits have also been recorded with repeated annual PRP injections [41]. A systematic review by Chalidis et al. [10] revealed that PRP injections are a safe an effective treatment option for early and moderate knee OA and its combination with radiofrequency ablation could have additional functional benefits and a greater reduction in VAS score. Other publications have further noted greater benefits in younger patients, potentially due to higher anabolic growth factor concentrations and a greater proportion of viable chondrocytes in younger cartilage [24,42,43]. Platelet-rich plasma augmentation of the microfracture technique has been shown also to accelerate and prolong the therapeutic effect in chondral lesions, although differences versus microfracture alone were not always statistically significant [41]. These findings suggest that PRP may serve not only as a stand-alone therapy but also as a valuable adjunct to surgical cartilage restoration techniques.

The main strengths of this review are the inclusion of prospective and randomized studies that used validated clinical and imaging outcomes, appropriate control groups, and consistent adverse event monitoring. Limitations of the systematic review include the heterogeneity of the applied PRP protocols, the small sample sizes, the short follow-up (less than a year), the inconsistent use of advanced imaging, the lack of blinding and inclusion of control group in some studies. Most included studies were below the minimum sample size required for adequate statistical power. Consequently, they were mainly capable of detecting large effects, and smaller but clinically relevant benefits of PRP may have been underestimated. The absence of subgroup analyses by age, sex, disease severity, or prior injuries underscores the importance of more precise patient classification in future trials. In addition, heterogeneity in PRP preparation and administration—including leukocyte content, platelet concentration, activation methods, injection volume, and dosing schedules—impedes comparability across studies. These limitations affect the generalizability and reproducibility of the results and highlight the need for the development of international consensus guidelines to ensure the highest standard of care for all patients suffering from PF disorders. Therefore, and despite the encouraging results, the current evidence—limited by small sample sizes, short follow-up, and lack of multicenter randomized trials—does not yet justify recommending PRP as a first-line treatment for patellofemoral joint disorders.

## 5. Conclusions

In conclusion, the current literature indicates that PRP is a safe intervention with a therapeutic potential for patients with CMP, PFA, and AKP, but its effectiveness and clinical impact require further research. Advanced MRI sequences have shown that PRP may also have a benefit on cartilage matrix quality, but evidence of cartilage regeneration is still lacking. Further well-designed, larger-scale randomized controlled trials with standardized PRP protocols, and longer follow-up are deemed necessary to better define patient selection criteria, optimize injection regimens, and clarify the role of imaging biomarkers in the assessment of cartilage repair and regeneration.

## Figures and Tables

**Table 1:** Level of evidence, quality, and risk of bias assessment of the included studies.

Author	Year	Type of Study	Level of Evidence	Quality of Study	Risk of Bias	Size Effects
Cobianchi Bellisari et al. [21]	2021	Retrospective comparative study	III	Moderate	Moderate	90%
Gürsoy et al. [22]	2025	Retrospective comparative study	III	Moderate	Moderate	80%
Örsçelik et al. [23]	2020	Randomized controlled trial	I	Moderate	Some concerns	90%
Ostojic et al. [24]	2024	Prospective comparative study	II	Moderate	Moderate	80%
Pintat et al. [25]	2017	Prospective cohort study	II	Moderate	Moderate	60%

**Table 2:** Characteristics of the eligible studies and patients.

Study (Author, Year)	Study Type	NoP Having PRP Injection	Age Range (Years)	Follow-Up (Months)	Type of Injection	Control Group (NoP)
Cobianchi Bellisari et al., 2021 [21]	Retrospective comparative study	34	22–54	12	PRP (3×, 8–10 mL, 3 weeks apart)	Conservative treatment (34)
Gürsoy et al., 2025 [22]	Retrospective comparative study	27	18–40	6	PRP (2×, 5cc, 2 weeks apart)	Conservative treatment (28), asymptomatic control group (25)
Örsçelik et al., 2020 [23]	Randomized controlled trial	38	21–66	12	PRP (3×, intra-articular, 3 weeks apart)	Prolotherapy (31)
Ostojic et al., 2024 [24]	Prospective comparative study	28	18–50	3 and 6	PRP (3×, 4–6 mL, 7–10 days apart) + HA (with 2nd PRP injection)	Physiotherapy (15)
Pintat et al., 2017 [25]	Prospective cohort study	19	27–57	12	PRP + MSC (single inj.)	None

Abbreviations: HA = hyaluronic acid; MSC = mesenchymal stem cells; NoP: number of patients; PRP = platelet-rich plasma.

**Table 3:** Mean values of patient-reported outcome measures of the included studies.

Study	VAS Baseline	VAS Final	WOMAC Baseline	WOMAC Final	Oxford Baseline	Oxford Final	Kujala Baseline	Kujala Final	Tegner/Lysholm Baseline	Tegner/Lysholm Final
Cobianchi Bellisari et al. [21]	7 (IQR: 6.0–7.2)	2 (IQR: 1.7–3.0)	18.3 ± 4.5	7.3 ± 3.2						
Gürsoy et al. [22]	7.1 ± 0.9	4.1 ± 1.7			35.1 ± 4.5	37.4 ± 5.0			66.6 ± 9.9	78.2 ± 12.3
Örsçelik et al. [23]	7.5	1							64.1	94.5
Ostojic et al. [24]	4.8	1.7					71	83		
Pintat et al. [25]			34.3 ± 24	14.1 ± 14.2						

Abbreviations: IQR: interquartile range; VAS: Visual Analog Scale; WOMAC: Western Ontario and McMaster Universities Osteoarthritis Index.

## Abbreviations

The following abbreviations are used in this manuscript:

AKP	Anterior knee pain
CI	Confidence interval
CMP	Chondromalacia patellae
HA	Hyaluronic acid
IQR	Interquartile range
MD	Mean difference
KOOS	Knee injury and osteoarthritis outcome score
MSC	Mesenchymal stem cells
NoP	Number of patients
OA	Osteoarthritis
PFA	Patellofemoral arthritis
PRP	Platelet-rich plasma
VAS	Visual Analog Scale
WOMAC	Western Ontario and McMaster Universities Osteoarthritis Index
